# Identification and verification of feature biomarkers associated with immune cells in neonatal sepsis

**DOI:** 10.1186/s40001-023-01061-2

**Published:** 2023-02-28

**Authors:** Weiqiang Liao, Huimin Xiao, Jinning He, Lili Huang, Yanxia Liao, Jiaohong Qin, Qiuping Yang, Liuhong Qu, Fei Ma, Sitao Li

**Affiliations:** 1 Department of Pediatrics, Dongguan Houjie Hospital, Dongguan, 523945 China; 2 Department of Pediatrics, The Sixth Affiliated Hospital, Sun Yat-Sen University, Guangzhou, 510655 China; 3 Department of Neonatology, The Maternal and Child Health Care Hospital of Huadu, Guangzhou, 510800 China; 4 Department of Neonatology, Maternal and Child Health Research Institute, Zhuhai Women and Children’s Hospital, Zhuhai, 519001 China

**Keywords:** Neonatal sepsis, Immune infiltration, Diagnosis model, Biomarkers, Logistic regression

## Abstract

**Background:**

Neonatal sepsis (NS), a life-threatening condition, is characterized by organ dysfunction and is the most common cause of neonatal death. However, the pathogenesis of NS is unclear and the clinical inflammatory markers currently used are not ideal for diagnosis of NS. Thus, exploring the link between immune responses in NS pathogenesis, elucidating the molecular mechanisms involved, and identifying potential therapeutic targets is of great significance in clinical practice. Herein, our study aimed to explore immune-related genes in NS and identify potential diagnostic biomarkers. Datasets for patients with NS and healthy controls were downloaded from the GEO database; GSE69686 and GSE25504 were used as the analysis and validation datasets, respectively. Differentially expressed genes (DEGs) were identified and Gene Set Enrichment Analysis (GSEA) was performed to determine their biological functions. Composition of immune cells was determined and immune-related genes (IRGs) between the two clusters were identified and their metabolic pathways were determined. Key genes with correlation coefficient > 0.5 and *p* < 0.05 were selected as screening biomarkers. Logistic regression models were constructed based on the selected biomarkers, and the diagnostic models were validated.

**Results:**

Fifty-two DEGs were identified, and GSEA indicated involvement in acute inflammatory response, bacterial detection, and regulation of macrophage activation. Most infiltrating immune cells, including activated CD8 + T cells, were significantly different in patients with NS compared to the healthy controls. Fifty-four IRGs were identified, and GSEA indicated involvement in immune response and macrophage activation and regulation of T cell activation. Diagnostic models of DEGs containing five genes (*PROS1, TDRD9, RETN, LOC728401, and METTL7B*) and IRG with one gene (*NSUN7*) constructed using LASSO algorithm were validated using the GPL6947 and GPL13667 subset datasets, respectively. The IRG model outperformed the DEG model. Additionally, statistical analysis suggested that risk scores may be related to gestational age and birth weight, regardless of sex.

**Conclusions:**

We identified six IRGs as potential diagnostic biomarkers for NS and developed diagnostic models for NS. Our findings provide a new perspective for future research on NS pathogenesis.

**Supplementary Information:**

The online version contains supplementary material available at 10.1186/s40001-023-01061-2.

## Background

Sepsis is a life-threatening organ dysfunction caused by a dysregulated host response to infection, mainly manifested as an inflammatory response and immunosuppression, and is currently the main cause of death in critically ill patients worldwide [1]. In the US, the present incidence of sepsis is approximately three per thousand, and severe sepsis kills at least 200,000 people annually [2]. Severe sepsis and septic shock account for 30–50% of hospital-reported deaths around the world [3]. Neonatal sepsis (NS) refers to bacteraemia with systemic infection occurring within the first month of life [4]. It is the most common cause of neonatal death, and its associated mortality is currently a major health concern worldwide [5]. NS can be divided into early- and late-onset, with 72 h after birth as the demarcation between the two. Neonatal infections account for an estimated 26% of under-five deaths [6]. In low- and middle-income countries, the reported incidence of NS in 2022 was 17.7% (5425/30577) and the mortality rate was 16.2% (877/5425) [7]. Development of primary and secondary prevention strategies based on different types of infections has become a hot area of NS-related research in recent decades [8].

Immune and inflammatory responses play important roles in the pathogenesis of NS. Currently, the commonly used clinical inflammatory markers are interleukin-6 (IL-6), C-reactive protein (CRP), and procalcitonin (PCT). IL-6 is a cytokine produced by mononuclear phagocytes, endothelial cells, fibroblasts, and decidual, chorionic, amniotic, and trophoblast cells upon stimulation with microbial products [9]. CRP, a protein synthesized in the liver, is currently used as an important biomarker to assess the severity and prognosis of NS [10]. PCT is produced by the parathyroid and neuroendocrine cells and acts as a precursor of calcitonin, which was formally proposed as a diagnostic marker for NS [11–13] in 2008 and can increase more than 1000-fold during active infection. However, these are not ideal for the diagnosis and prognosis of NS [14]. In the early stages of NS, various immune cells (such as monocytes and macrophages) and released inflammatory mediators and cytokines can induce an excessive inflammatory response, whereas in the late stage, immunosuppression is predominant [15, 16]. Exploring the link between immune responses in the pathogenesis of NS, elucidating the molecular mechanisms involved, and identifying potential therapeutic targets will be of great significance in clinical practice.

Bioinformatic analysis helps to understand the underlying mechanisms of NS by screening gene expression datasets. In the present study, differentially expressed genes (DEGs) between NS and healthy controls were identified through bioinformatic analysis, and the underlying pathology of NS was explored through detection of the immune microenvironment, clustering, and protein–protein network analysis. In addition, we constructed a diagnostic model of six identified DEGs using least absolute shrinkage and selection operator (LASSO) regression analysis. Finally, we confirmed the effectiveness of the diagnostic model of immune-related genes (IRGs) using the GSE25504 dataset. In the present study, we explored the pathogenesis of NS from the perspectives of immunity and inflammation, which can identify potential targets for treating NS.

## Results

### Gene expression features of NS samples

**Fig. 1:**
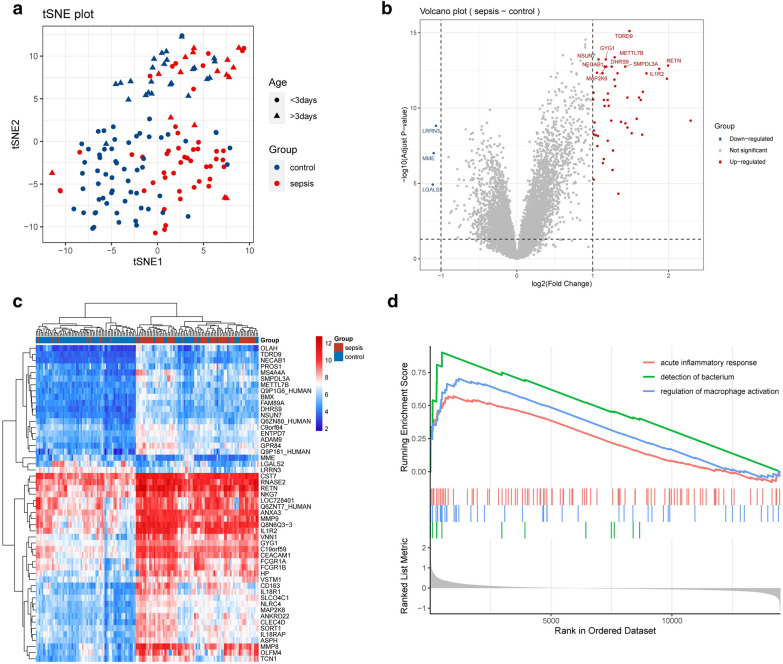
Gene expression characteristics in neonatal sepsis (NS) samples. **a** Dimension reduction algorithm was used to evaluate the differences between patients with NS and normal samples. **b** The differentially expressed genes (DEGs) in total RNA expression profiles between NS and normal samples were visualized by Vioplot. **c** Heatmaps presented the expression of all DEGs. **d** Gene Set Enrichment Analysis (GSEA) analysis was performed to evaluate the differences of the biological states between NS and normal samples

The DEGs between NS and normal samples are shown in Fig. 1. t-Distributed stochastic neighbour embedding (t-SNE) was conducted to evaluate the differences in gene expression between NS and normal samples (Fig. 1a, see Additional file 1: Table S1). Compared with normal samples, 52 DEGs were found in NS samples, most of which were up-regulated (Fig. 1b). Heatmaps were conducted to visualize the 52 DEGs (Fig. 1c). Gene Set Enrichment Analysis (GSEA) analysis was also carried out to explore the functional pathways between NS and normal samples, and the results indicated that the DEGs were considerably enriched in acute inflammatory response, detection of bacterium, and regulation of macrophage activation (Fig. 1d).

### Immunological characteristics of NS samples

**Fig. 2:**
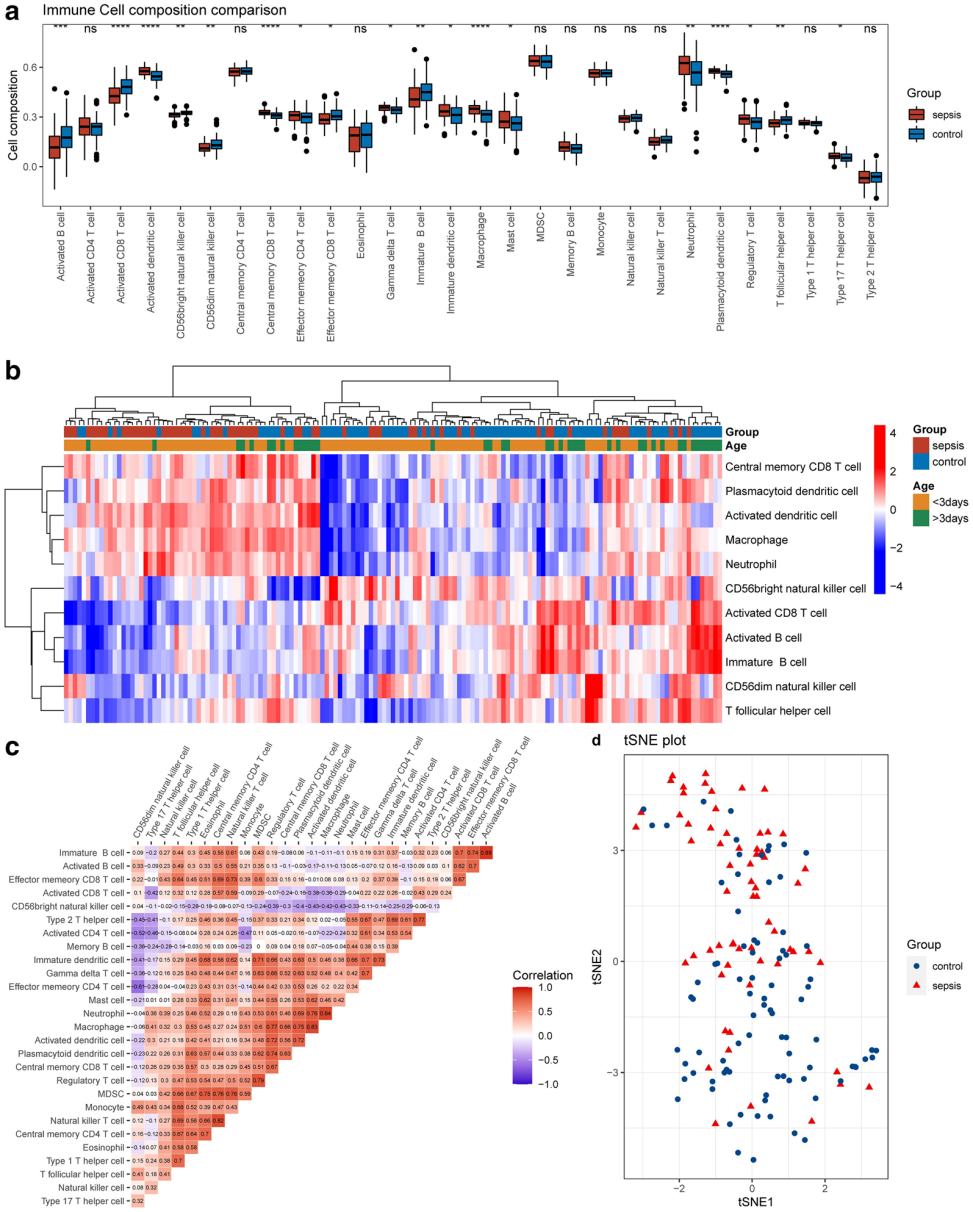
Characteristics of the immune cell microenvironment in NS. **a** Differences in immune cell compositions between NS and normal samples. **b** The differences of immune cell compositions between NS and normal samples were visualized by heatmap; grouped by age. **c** The correlation of the immune cells was visualized by corrplot. **d** Dimension reduction algorithm was conducted to evaluate the differences in immune cell compositions between NS and normal samples. t-SNE, t-distributed stochastic neighbour embedding

To explore the immune microenvironment in patients with NS, the concentration of immune cells was quantified. As shown in Fig. 2a, most of the infiltrating immune cells showed significant differences in patients with NS, which was also demonstrated by hierarchical clustering analysis (Fig. 2b) and t-SNE plot (Fig. 2d). In addition, the concentration of most of the immune cells were considerably correlated (Fig. 2c). The all detected immune cells see Additional file 2: Table S2.

**Fig. 3:**
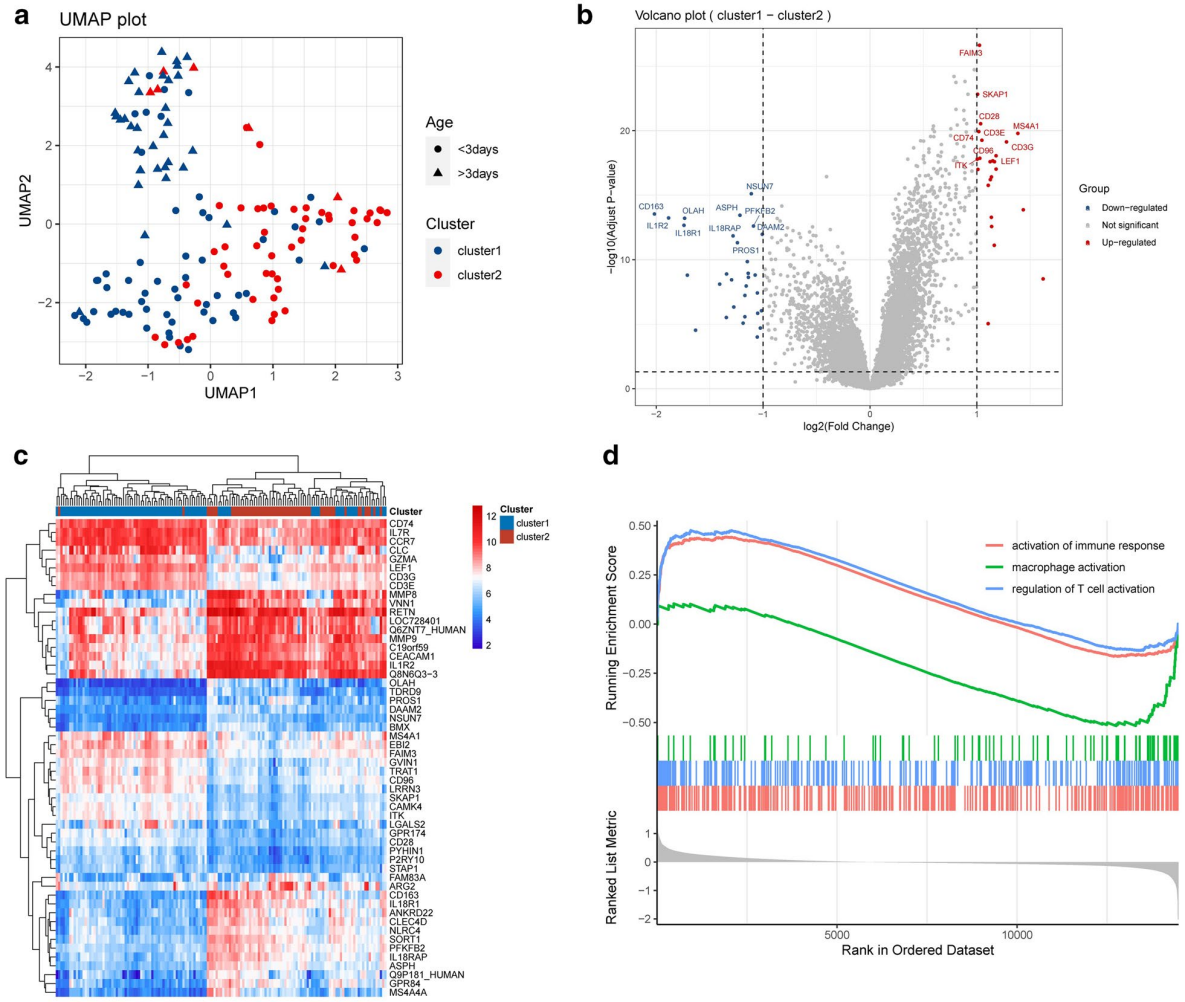
Gene expression characteristics of the two immune-related clusters. **a** Dimension reduction algorithm was used to evaluate the differences between cluster 1 and cluster 2. **b** Immune-related DEGs (IRGs) of total RNA expression profile between cluster 1 and cluster 2 were visualized by Vioplot. **c** Heatmaps presented the expression of all IRGs. **d** GSEA was performed to evaluate the differences of the biological states between cluster 1 and cluster 2

The IRGs in patients with NS were further explored. UMAP plot showed that there were significant differences in gene signatures between the two clusters divided by immune cell composition (Fig. 3a). Compared with normal samples, 54 IRGs were found, with 30 down-regulated and 24 up-regulated genes (Fig. 3b, see Additional file 3: Table S3). Heatmaps were generated to visualize IRG expression in the two clusters (Fig. 3c). GSEA was carried out, and the results indicated that the functions of IRGs were considerably enriched in activation of immune response, macrophage activation, and regulation of T cell activation (Fig. 3d).

### Significance of gene expression signatures in NS diagnosis

**Fig. 4:**
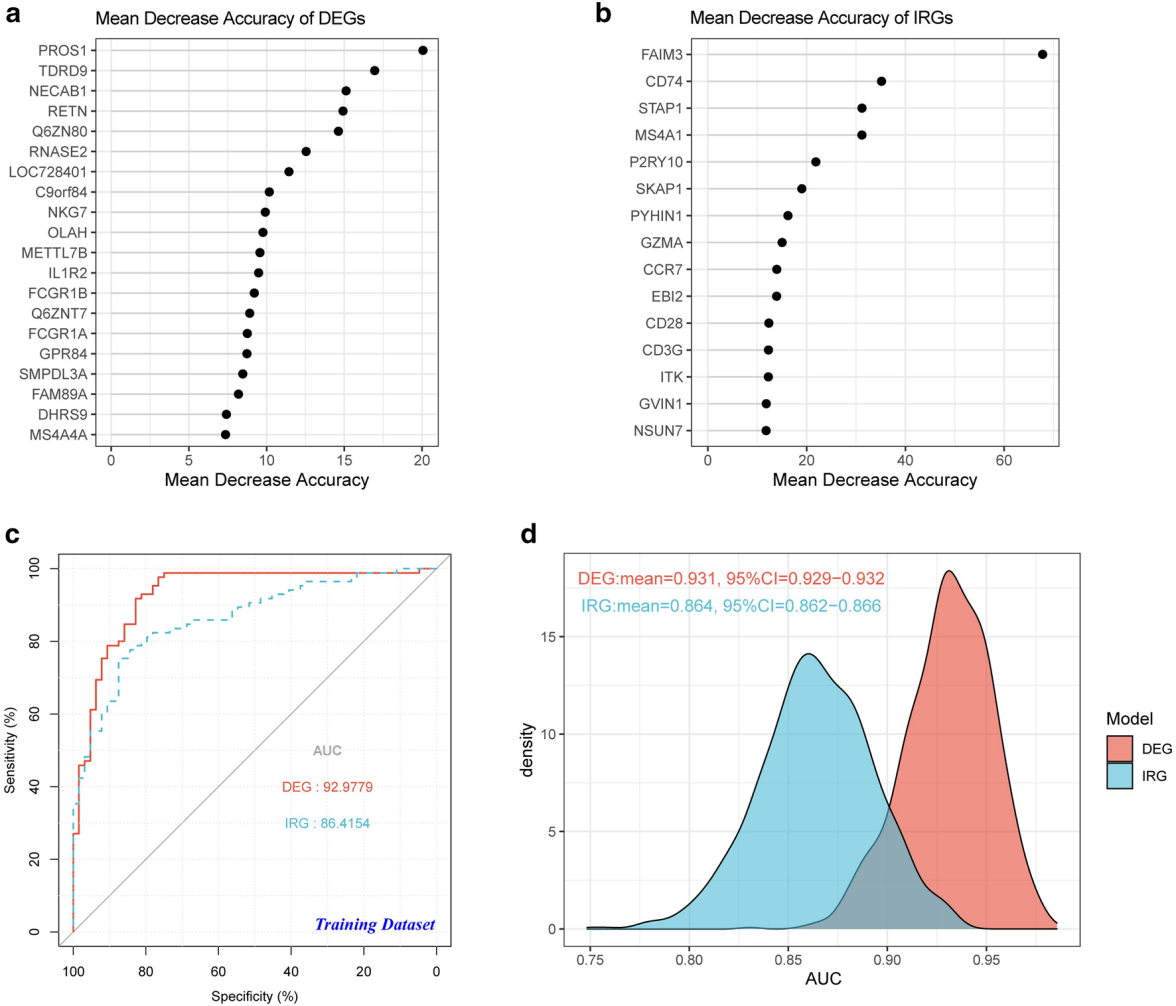
Construction of NS diagnostic models based on DEGs and IRGs. **a** Top 20 DEGs sorted by mean decrease accuracy based on random forest method. **b** Top 15 IRGs sorted by mean decrease accuracy based on random forest method. **c** Receiver operating characteristic (ROC) curves were calculated to evaluate the diagnostic efficiency of the DEG and IRG gene signatures with the training dataset. **d** AUC values of both models obtained by 1000 repeated tests based on bootstrap methods were shown in the density plot to validate the conclusions. AUC, area under the curve; CI, confidence interval; DEG, differentially expressed gene; IRG, immune-related gene

After filtering gene signatures with random forest method, 20 DEGs and 15 IRGs were used to build the diagnostic models, as shown in Fig. 4a and b. LASSO algorithm was used to construct a diagnostic model to classify the training dataset into NS and control groups. Two diagnostic models were built, respectively, with DEG and IRG signatures (see Additional file 4: Table S4, Additional file 5: Table S5). For the DEG model, 5 regulators (*PROS1, TDRD9, RETN, LOC728401*, and *METTL7B*) and corresponding coefficients were identified with minimum fivefold cross-validated mean square error in GSE69686. For the IRG model, there was only one regulator *NSUN7*. The risk score for each patient was calculated as the product of coefficient and the sum of gene expression. As shown in Fig. 4c, the risk scores of gene signatures could robustly predict diagnosis for patients in both models. Additionally, bootstrap method was adopted to confirm the robustness of the two diagnostic models. The results of 1000 repeated tests are shown in Fig. 4d.

**Fig. 5:**
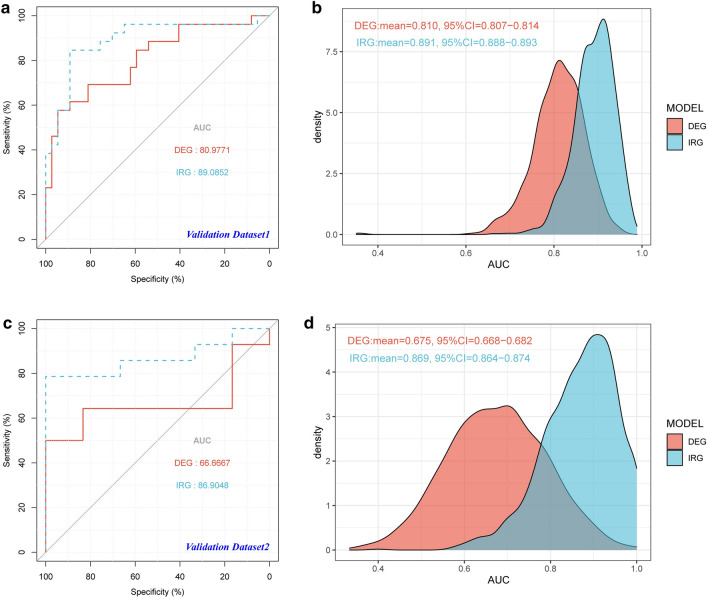
Evaluation of NS diagnostic models based on DEGs and IRGs. **a** ROC curves were calculated to evaluate the diagnostic efficiency of the DEG and IRG gene signatures with the validation dataset 1. **b** AUC values of both models obtained by 1000 repeated tests based on bootstrap methods were shown in the density plot to validate the conclusions. **c** ROC curves were calculated to evaluate the diagnostic efficiency of the DEG and IRG signatures with the validation dataset 2. **d** AUC values of both models obtained by 1000 repeated tests based on bootstrap methods were shown in the density plot to validate the conclusions. *AUC* area under the curve, *CI* confidence interval, *DEG* differentially expressed gene, *IRG* immune-related gene

In addition, we evaluated the effectiveness of the two diagnostic models in the validation dataset GSE25504 (platform GPL6947 as validation dataset 1 and platform GPL13667 as validation dataset 2). It should be noted that the DEG model’s regulator LOC728401 is missing in both validation datasets; however, the coefficient is much smaller than other regulators (about 1/5) and could be ignored. Receiver operating characteristic (ROC) curve and bootstrap methods were used again (Fig. 5). The results showed that both models were applicable to validation dataset 1 (Fig. 5a, b), and the IRG model was more robust than the DEG model in validation dataset 2, because it had only one gene signature (Fig. 5c, d).

Finally, the relationship between risk scores of the diagnostic models and phenotype in the validation datasets was analysed (Table 1). The results showed that the risk scores were probably related to gestational age and birthweight and not to sex.

**Table 1:** Relationship between risk scores of both diagnostic models and phenotype in the two validation datasets

Validation dataset 1 (DEG model)			
Level	High risk	Low risk	*P* value
	*n* = 31	*n* = 32	
Group (%)			
Control	13 (41.9)	24 (75.0)	0.011
Infected	18 (58.1)	8 (25.0)	
Sex (%)			
Female	13 (41.9)	13 (40.6)	1
Male	18 (58.1)	19 (59.4)	
Corrected gestational age (mean (SD))	236.90 (36.03)	262.69 (35.67)	0.006
Birthweight (mean (SD))	1863.29 (1233.33)	2593.25 (1389.79)	0.031

Validation dataset 1 (IRG model)			
Level	High risk	Low risk	*P* value
	*n* = 31	*n* = 32	
Group (%)			
Control	9 (29.0)	28 (87.5)	< 0.001
Infected	22 (71.0)	4 (12.5)	
Sex (%)			
Female	14 (45.2)	12 (37.5)	0.613
Male	17 (54.8)	20 (62.5)	
Corrected gestational age (mean (SD))	223.94 (28.09)	275.25 (27.65)	< 0.001
Birthweight (mean (SD))	1394.23 (929.43)	3047.66 (1204.01)	< 0.001

Validation dataset 2 (DEG model)			
Level	High risk	Low risk	*P* value
	*n* = 10	*n* = 10	
Group (%)			
Control	1 (10.0)	5 (50.0)	0.141
Infected	9 (90.0)	5 (50.0)	
Sex (%)			
Female	2 (20.0)	2 (20.0)	1
Male	8 (80.0)	8 (80.0)	
Corrected gestational age (mean (SD))	242.50 (18.74)	234.40 (25.98)	0.434
Birthweight (mean (SD))	1344.50 (309.98)	1029.50 (385.61)	0.059

Validation dataset 2 (IRG model)			
Level	High risk	Low risk	*P* value
	*n* = 10	*n* = 10	
Group (%)			
Control	0 (0.0)	6 (60.0)	0.011
Infected	10 (00.0)	4 (40.0)	
Sex (%)			
Female	4 (40.0)	0 (0.0)	0.087
Male	6 (60.0)	10 (100.0)	
Corrected gestational age (mean (SD))	244.20 (24.56)	232.70 (19.64)	0.263
Birthweight (mean (SD))	1156.00 (348.22)	1218.00 (420.14)	0.724

*DEG* differentially expressed gene, *SD* standard deviation, *IRG* immune-related gene

## Discussion

NS, a life-threatening condition, can lead to microcirculatory disturbances, immune dysfunction, and tissue and organ dysfunction, and is becoming the most common cause of neonatal death worldwide [4]. Hence, NS and its related mortality and complications represent a major global health concern [2–6].

Impaired inflammatory immune responses during the onset and recovery phases are considered a hallmark of severe NS. Abnormal activation of macrophages and neutrophils occurs in the early stage of NS [17], and the recovery period is mainly characterized by immunosuppression. Sepsis is characterized by upregulation of CD4 + and CD8 + T cells, T helper 17 cells, and regulatory T cells [16], lymphopenia, and loss of immune function. Microarray analysis has indicated abnormalities in the expression of immune-related genes in children with sepsis, including *FYN, FBL, ATM, WDR75, FOXO1,* and *ITK* [18]. Alterations in gene expression related to innate immunity have also been reported in NS [19, 20]. The innate immune response in NS is driven by genes involved in innate immunity, such as *IL1R2, ILRN,* and *SOCS3* [21]. The risk of developing NS is also associated with polymorphisms in exon 1 of mannose-binding lectin and Toll-like receptor 4 [22]. Based on the immunomodulatory effects of rhIL-7 in sepsis [23], targeting T cell immunometabolism in early or late sepsis has great therapeutic potential [16]. However, the pathogenesis of NS has not yet been fully established and needs further understanding.

In the present study, bioinformatic analysis and GSEA of DEGs in the merged dataset showed significant enrichment of immune and inflammatory responses, including acute inflammatory response, bacterial detection (including coagulase-negative Staphylococcus, Enterococcus species, et al. [19, 24]), and regulation of macrophage activation, which play important roles in the pathogenesis of NS. Most infiltrating immune cells were significantly different in patients with NS compared to the control group; activated CD8 + T and B cells, CD56 natural killer cells, naïve dendritic cells, and T helper cells were significantly enriched in the sepsis group, whereas activated dendritic cells, memory CD8 + T cells, macrophages, plasmacytoid dendritic cells, and neutrophils were significantly enriched in the control group. GSEA of IRGs showed that their functions were significantly enriched in the activation of immune response, macrophage, and the regulation of T cells. The diagnostic model of DEG containing five genes (*PROS1, TDRD9, RETN, LOC728401*, and *METTL7B*) and that of IRG with one gene (*NSUN7*) were constructed using LASSO algorithm, and their diagnostic performance verified by correlation and logical analyses showed good area under the curve (AUC) scores. Additionally, the DEG and IRG models were verified in the GPL6947 and GPL13667 sub-datasets, respectively. The IRG model performed better than the DEG model. The IRG model contained only *NSUN7* suggesting that this gene may be important for the diagnosis and treatment of NS. Finally, statistical analysis of the validation datasets suggested that the risk scores may be related to gestational age and birth weight, regardless of sex.

Current knowledge of human B and T cells in sepsis is sparse, discordant, and at variance with findings reported from animal models. Our research find the activated B cell and activated CD8 T cells showed lesser expression in sepsis cases compared to control. These data are in agreement with those published in previous studies. Hotchkiss et al. [25] demonstrated that patients with sepsis show a severe B-cell deficiency. Monserrat et al. [17] pointed that B-cell lymphopenia affects the B-cell subsets heterogeneously, with marked reduction of CD19 + CD23 + B cells (activated regulatory B cells) and CD19 + CD5 + B cells (natural responder B-1a cells), but with normal numbers of CD19 + CD69 + early activated B cells. Similar findings were reported by other groups [26]. Meanwhile it is established that septic shock is associated with a severe exhaustion and depletion of T lymphocytes [27]. So the present results establish an association between decreased lymphocytes and sepsis but do not establish causality between lymphocyte apoptosis and outcome in patients with sepsis, which required further investigation.

Sun RNA methyltransferase 7 (*NSUN7*) belonging to the methyltransferase superfamily is located on chromosome 4p14 and consists of 12 exons and 718 amino acids. It reduces protein activity and motility of sperms and is associated with male infertility [28]. High expression of NSUN7 is associated with shortened survival in low-grade gliomas [29]. The overall survival in Ewing sarcoma is significantly associated with NSUN7 immunoreactivity, an independent favourable prognostic marker [30]. *NSUN7* may also serve as a pivotal biomarker for predicting biochemical recurrence in patients with prostate cancer [31]. An increase in the mean precursor strength of plasma protein polypeptides, such as *NSUN7,* is associated with sepsis [32]. *NSUN7* may also be associated with psychiatric disorders, including schizophrenia, bipolar disorder [33], and major depressive disorders. In eukaryotes, the *NSUN* family is the major RNA m5C modifying enzyme and includes seven family members (*NSUN1–7*). The biological function and significance of RNA m5C modification in maintaining mRNA stability is essential during early embryonic development and in the post-embryonic immune system. *NSUN7* has been systematically studied in male sperm motility, but its mechanism of action in tumours and sepsis has not been elucidated. In the present study, *NSUN7* expression was up-regulated in the NS group. Combined with bioinformatic analyses, *NSUN7* may be used as a biomarker for the pathogenesis of NS.

Resistin (*RETN*), located on chromosome 19p13.2, encodes an anti-retro-transcriptional protein and belongs to the resistance protein-like gene family. Its encoded protein, a 114 amino acid polypeptide (12.5 kDa) hormone, is secreted by adipocytes and is a member of the cysteine-rich small secreted protein gene family [34, 35]. RETN activates monocytes and macrophages and induces the release of proinflammatory cytokines including lipopolysaccharides, IL-1, IL-6, and tumour necrosis factor (TNF)-α [36–38]. RETN promotes endothelial cell activation and smooth muscle cell proliferation [39]. Elevated RETN levels have been reported in sepsis samples [40–43]. Clinical observations have indicated that plasma RETN levels are highly correlated with the levels of inflammatory markers, such as CRP and IL-6 [44]. Additionally, RETN increases endothelial cell permeability, thereby promoting the adhesion and infiltration of endothelial cells and monocytes. RETN also mediates immunosuppression, directly suppresses neutrophil function, and is associated with poor outcomes in sepsis [45]. These findings suggest a link between RETN, immunity, and inflammation. In the present study, RETN expression was up-regulated in the NS group, indicating that *RETN* may be involved in the occurrence and development of NS.

Protein S1 (*PROS1*), located on chromosome 3q11.1, is a vitamin K-dependent plasma protein that activates coagulation factors V and VIII by activating protein C while promoting the clearance of early apoptotic cells [46]. Tyrosine kinase receptor (TAM receptor) regulates the basic mediator of inflammatory response; PROS1 acts as a ligand of TAM receptor; and the expression of proinflammatory factors, such as TNF-α and CCL3, is increased during *PROS1* deficiency [45]. PROS1 expression is positively correlated with neutrophil count and activity and oxidative burst, and is a potential therapeutic target for decompensated cirrhosis and sepsis [46]. PROS1 can be used as a targeted drug for the treatment of inflammatory diseases, such as spinal cord injury and ankylosing spondylitis [47]. In the present study, *PROS1* expression was up-regulated in the NS group. The role of *PROS1* in the coagulation mechanism has been systematically studied; however, its role in NS has not been elucidated.

Methyltransferase 7B (METTL7B) belongs to the methyltransferase-like protein family, and is located on chromosome 12. To date, the function of *METTL7B* is unclear, although several studies have linked it to specific disease states, subcellular localization, and cellular processes [48, 49]. A recent study found that *METTL7B* has methylase activity, which can methylate intracellular alkanethiol molecules and reduce associated cellular toxicity [49, 50]. METTL7B expression is associated with immune cells, such as B cells, CD4 + T cells, CD8 + T cells, monocytes, neutrophils, macrophages, and activated mast cells. Clinical studies have shown that *METTL7B* responds to inflammatory signals via Janus Kinase 1 [51]. In the present study, *METTL7B* expression was up-regulated in the NS group, indicating that *METTL7B* may be involved in the occurrence and development of NS.

Tudor domain-containing protein 9 (*TDRD9*) is a DEXH-box RNA helicase, which is involved in PIWI-interacting RNA formation [52]. *TDRD9* is a DNA damage and repair-associated gene and is mainly expressed in sperms [53]. It can be used to predict disease-free survival in cancers, such as clear cell renal cell carcinoma and thyroid cancer [54, 55]. In addition to the male reproductive system, it is mainly expressed in the blood cells, including monocytes and dendritic cells, which play important roles in the innate immune response [56].

The novelty of our study is as follows. First, we used bioinformatic analysis to investigate the molecular mechanisms of NS from the perspectives of immunity and inflammation. Second, we found that NSUN7, PROS1, TDRD9, RETN, LOC728401, and METTL7B may be potential diagnostic biomarkers for NS, particularly NSUN7. However, this study has some limitations. First, we could not determine whether a causal relationship exists between the differences in gene expression and pathophysiological mechanisms of NS or if it is simply a compensatory change. Second, the study was a retrospective data analysis; therefore, we lacked detailed clinical and prognostic data, which limited further exploration of the genes for their clinical characteristics and outcomes. Finally, our study was based on bioinformatic analysis of transcriptome data from public datasets, which may be inconsistent with the actual situation. Further clinical trials are needed to validate our findings.

## Conclusions

Through bioinformatic analysis of published transcriptional data, NSUN7, PROS1, TDRD9, RETN, LOC728401, and METTL7B were identified as potential biomarkers of NS from the perspective of immune cell infiltration combined with logistic regression. More importantly, the developed diagnostic models provide a new perspective for future research on the pathogenesis of NS.

## Methods

### NS datasets and data process

RNA sequencing data that investigated gene expression in peripheral blood samples from patients with NS were downloaded from the Gene Expression Omnibus (GEO) database, which included GSE69686 (including 64 NS and 85 control samples), and GSE25504 (including 170 samples, which were divided into four platforms, involving GPL570, GPL6947, GPL13667, and GPL15158). In consideration of sample size and sequencing platforms, GSE69686 was used as analysis dataset and GSE25504 (GPL13667 and GPL6947 platform) was used as validation datasets. Next, the corresponding expression matrix and clinical information were download and matched. The expression matrix were pre-processed via quantile normalization with R package limma [57].

### Identifying DEGs between NS and normal samples

In order to identify DEGs, the R package limma [1] which implements an empirical Bayesian approach to estimate gene-expression changes using moderated t-tests, was applied to determine DEGs among different groups; DEGs were screened by criteria (adjusted *P* value < 0.05) as implemented in limma. Volcano plots were generated to visualize the expression of DEGs. Hierarchal clustering was also conducted to measure the correlation of DEGs and identify potential gene modules by using R package pheatmap. In addition, to identify the potential function and involved pathways, we performed GSEA based on the differential expression profiles using the clusterProfiler R package [58].

### Depicting immunological characteristics of immune cell microenvironment in neonatal samples

The immunological characteristics of immune cell microenvironment in neonatal samples were depicted with the GSE69686 dataset. The Single-Sample Gene-Set Enrichment Analysis (ssGSEA) algorithm was used to quantify the relative abundance of tumour-infiltrating immune cells based on specific immune cell gene sets obtained from Charoentong et al. [59]. The differences between NS and normal samples were visualized with boxplots by using R package ggpubr, and the correlations among immune cells were shown in correlation heatmap.

### Unsupervised clustering by immune cell composition

To explore differences related with immune cell microenvironment between patients with NS and normal samples, we applied consensus clustering analysis to GSE69686 dataset based on the immune cell composition calculated by ssGSEA algorithm. This was performed using the Consensus Cluster Plus R package [60], and two subgroups were identified.

### Identifying IRGs between NS and normal samples

The R package limma was used to calculate IRGs between two clusters. Heatmap and volcano plots were generated to visualize the IRGs in two clusters. Furthermore, GSEA was performed based on IRGs to estimate related pathways.

### Gene expression signature identification and diagnostic model construction

DEGs and IRGs were used to build diagnostic models. Firstly, the random forest algorithm was used to filter genes used in model construction. According to the cross-validation results, the top 20 DEGs and top 15 IRGs sorted by mean decrease accuracy were selected (see Additional file 6: Fig. S6). Then, the LASSO algorithm was used to build classification models based on the actual diagnosis. At last, risk score of all samples was calculated according to the coefficients in the diagnostic models.

### Evaluating the effectiveness of diagnostic models

The effectiveness of the two diagnostic models was evaluated in the training dataset GSE69686 and validation datasets GSE25504 (GPL13667 and GPL6947 platform). ROC curve was used to evaluate the accuracy of the signatures in predicting the diagnostic results. In addition, bootstrap method was adopted to validate the reliability of ROC curve. The density plots showed the results of AUC calculated 1000 times for both datasets and models.

### Statistical analysis

Data were analysed with R (version 4.1.0) and R Bioconductor packages. Fisher’s exact test was used to analyse differences between high-risk and low-risk samples. *P*-values less than 0.05 were considered statistically significant.

## Supplementary Information

**Additional file 1: Table S1.** The DEGs between neonatal sepsis patients and normal samples.

**Additional file 2: Table S2.** The immune cell compositions of neonatal sepsis patients and normal samples.

**Additional file 3: Table S3.** The IRGs between neonatal sepsis patients and normal samples.

**Additional file 4: Table S4.** The coefficients of regulators in the DEG diagnostic model.

**Additional file 5: Table S5.** The coefficients of regulators in the IRG diagnostic model.

**Additional file 6: Figure S6.** Cross-validation error of classification with DEGs (a) and IRGs (b) based on random forest method.

## Data Availability

The datasets used and/or analysed during the current study are available from the corresponding author on reasonable request.

## Abbreviations

AUC: Area under the curve
CRP: C-reactive protein
DEGs: Differentially expressed genes
GSEA: Gene set enrichment analysis
IL: Interleukin
IRGs: Immune-related genes
LASSO: Least absolute shrinkage and selection operator
METTL7B: Methyltransferase 7B
NS: Neonatal sepsis
NSUN7: Sun RNA methyltransferase 7
PCT: Procalcitonin
PROS1: Protein S1
RETN: Resistin
ROC: Receiver operating characteristic
TDRD9: Tudor domain-containing protein 9
TNF: Tumour necrosis factor
t-SNE: T-distributed stochastic neighbour embedding
