# Supplementary figures and images for: Identification and verification of feature biomarkers associated with immune cells in neonatal sepsis
Source: Eur J Med Res. 2023 Feb 28;28:105. doi: 10.1186/s40001-023-01061-2 (PMC9972688; doi:10.1186/s40001-023-01061-2)

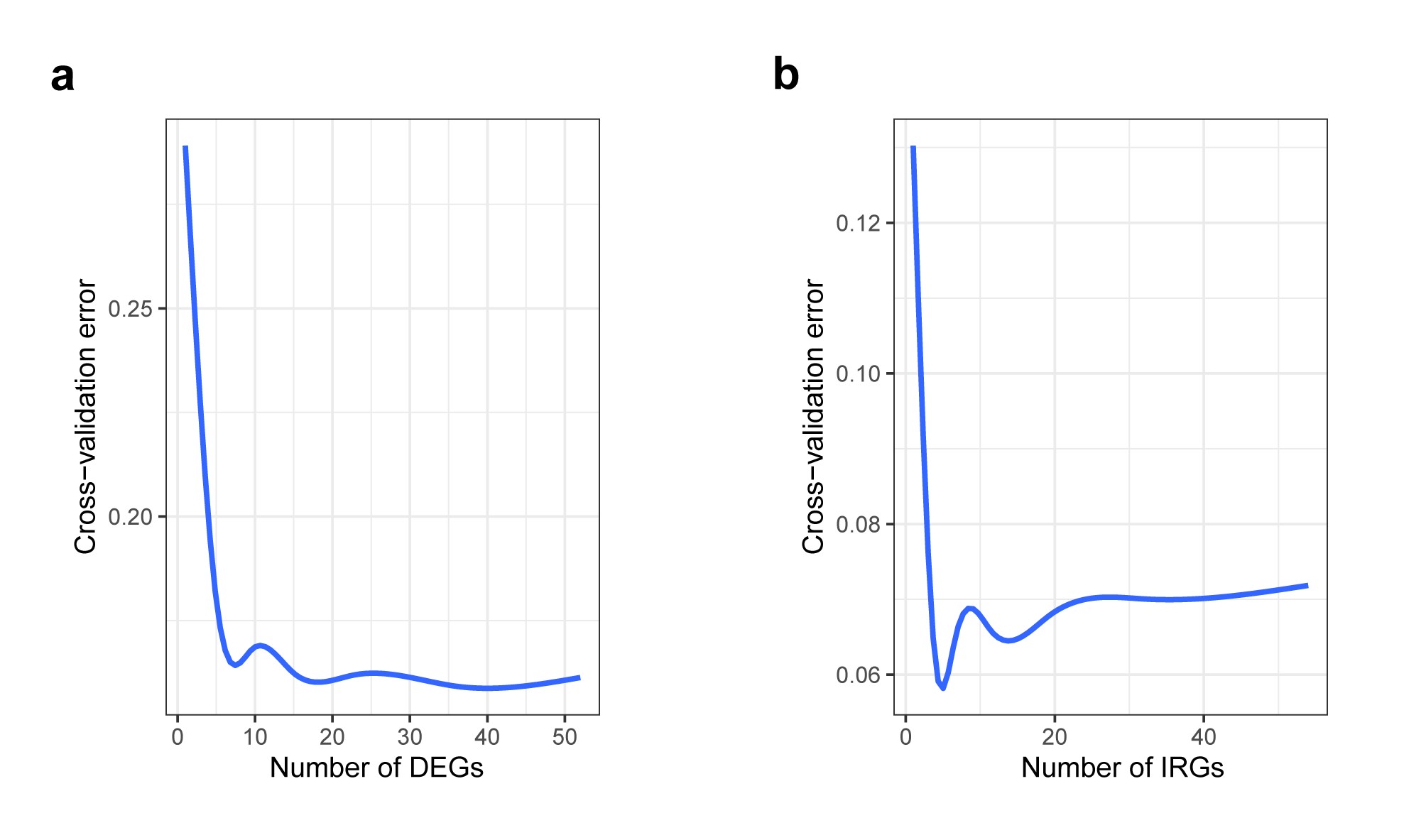

Supplement: Supplementary file 6 — Additional file 6: Figure S6. Cross-validation error of classification with DEGs (a) and IRGs (b) based on random forest method. [file 40001_2023_1061_MOESM6_ESM.jpg]
